# Mortality Patterns of Toxoplasmosis and Its Comorbidities in Tanzania: A 10-Year Retrospective Hospital-Based Survey

**DOI:** 10.3389/fpubh.2019.00025

**Published:** 2019-02-19

**Authors:** Leonard E. G. Mboera, Coleman Kishamawe, Evord Kimario, Susan F. Rumisha

**Affiliations:** ^1^Southern African Centre for Infectious Disease Surveillance, Chuo Kikuu, Morogoro, Tanzania; ^2^National Institute for Medical Research, Headquarters, Dar es Salaam, Tanzania; ^3^Mwanza Research Centre, National Institute for Medical Research, Mwanza, Tanzania

**Keywords:** toxoplasmosis, co-morbidity, mortality, hospital, Tanzania

## Abstract

**Introduction:** Toxoplasmosis is a parasitic zoonosis and an important cause of abortions, mental retardation, encephalitis, blindness, and death worldwide. Few studies have quantified toxoplasmosis mortality and associated medical conditions in Sub-Saharan Africa. This retrospective hospital-based study aimed to determine the mortality patterns of toxoplasmosis and its comorbidities among in-patients in Tanzania.

**Methods:** Data on causes of death were collected using customized paper-based collection tools. Sources of data included death registers, inpatient registers, and International Classification of Diseases report forms. All death events from January 2006 to December 2015 were collected. Data used in this study is a subset of deaths where the underlying cause of death was toxoplasmosis. Data was analyzed by STATA programme version 13.

**Results:** Thirty-seven public hospitals were involved in the study. A total of 188 deaths due to toxoplasmosis were reported during the 10-years period. Toxoplasmosis deaths accounted for 0.08% (188/247,976) of the total deaths recorded. The age-standardized mortality rate per 100,000 population increased from 0.11 in 2006 to 0.79 in 2015. Most deaths due to toxoplasmosis affected the adult age category. Of the 188 deaths, males accounted for 51.1% while females for 48.9% of the deaths. Dar es Salaam, Mbeya, Pwani, Tanga, and Mwanza contributed to over half (59.05%) of all deaths due to Toxoplasmosis. Of the total deaths due to toxoplasmosis, 70.7% were associated with other medical conditions; which included HIV/AIDS (52.6%), HIV/AIDS+Cryptococcal meningitis (18.8%) and HIV+Pneumocystis pneumonia (6.8%).

**Conclusion:** The age-standardized mortality rate due to toxoplasmosis has been increasing substantially between 2006 and 2015. Most deaths due to toxoplasmosis affected the adult age category and were highly associated with HIV/AIDS. Appropriate interventions are needed to alleviate the burden of toxoplasmosis in Tanzania.

## Introduction

Toxoplasmosis is a parasitic zoonosis and an important cause of abortions, mental retardation, encephalitis, blindness, and death worldwide (1). Globally, it is estimated that about one-third of the human population are infected with *Toxoplasma gondii* (1–3). It is the most common food-borne parasitic infection in high-income countries (4, 5). Globally, seroprevalence of *T. gondii* varies between 1 and 100% (2, 6–8).

Toxoplasma infections have been reported in both domestic and wild animals in Africa. *T. gondii* antibodies have been detected in zebra, hippopotamus, elephant, water buck, lion, and rock hyrax (9). Studies on the prevalence of anti-*T. gondii* antibody among domestic animals indicate that the overall prevalence to range from 12 to 37.4%. It is higher in chicken (37.4%), camels (36.0%), sheep (26.1%), and pigs (26.0%) and relatively lower in cattle (12.0%) (10). Human toxoplasmosis is reported to be widespread in Sub-Saharan Africa with a seroprevalence of 3.6–84% in different countries (6, 8, 11–14). The variation in the prevalence rates is attributed to the environmental and socio-cultural factors. The highest prevalence has been reported in areas where consumption of raw or undercooked meat is common and in areas where stray cats are abundant (2). However, the infection has remained undetected and hence, poorly managed due to inadequate diagnostic facilities (15).

Several studies have reported prevalence of toxoplasmosis in Tanzania, most of them focusing on pregnant women. Mwambe et al. (16) in their study in Mwanza reported that 30.9% of women were sero-positive for *T. gondii*-specific antibodies. In a study in Dar es Salaam a seroprevalence of 35% was reported among pregnant women (17). A survey carried out in Tanga district of north-eastern Tanzania reported that antibodies to *T. gondii* were detected in 46% of the individuals studied (18). *T. gondii* IgG and IgM seropositivities of 57.7 and 11.3%, respectively have been reported among pastoralists of northern Tanzania (19). Two studies at a tertiary hospital in northern Tanzania, reported that 41.7% (13) and 45% (20) of the expectant women were seropositive for *T. gondii*-specific antibodies.

Human infection can result from the ingestion or handling of undercooked or raw meat containing *Toxoplasma gondii* cysts. Toxoplasmosis is usually spread by eating poorly cooked food that contains cysts, exposure to infected cat feces, and vertically, from a mother to a child during pregnancy (21). Infection can also result from direct contact with cats or from the consumption of water or food contaminated by oocysts excreted in the feces of infected cats (22). Felines are the definitive hosts and so far are the only known animals capable of shedding the infective oocysts in the feces (23).

Few studies have quantified toxoplasmosis mortality and associated medical conditions (24, 25). However, such data are not available in most of the Sub-Saharan African countries including Tanzania despite *T. gondii* being an important zoonotic pathogen, and with high seroprevalence of the infection in both domestic animals and humans. This study aimed to determine the mortality pattern due to toxoplasmosis and its co-morbidities among in-patients in hospitals of Tanzania from 2006 to 2015.

## Materials and Methods

### Study Sites and Design

This retrospective study involved primary (district), secondary (regional referral), tertiary (national and zonal referral), and specialized hospitals in Tanzania. National, tertiary, and specialized hospitals were conveniently included in the study. A multistage sampling technique was employed to select the regional referral and district hospitals. Based on the population size, the country was divided into three main strata; namely highly populated regions (Dar es Salaam, Mwanza and Mbeya), medium populated (Kagera, Tabora, Morogoro, Kigoma, Dodoma, and Tanga), and lowly populated regions (Arusha, Geita, Iringa, Katavi, Kilimanjaro, Lindi, Manyara, Mara, Mtwara, Njombe, Pwani, Rukwa, Ruvuma, Shinyanga, Singida, and Simiyu). In the highly populated stratum, three hospitals were selected from each region; in medium populated two hospitals were selected from each region and from the lowly populated stratum, one hospital was selected from each region. In regions where a national or tertiary hospital was included, the regional hospital was left out. In addition, 10% of the district hospitals were included in the study. Other criteria considered to obtain a national representative sample were prevalence of malaria and HIV/AIDS, child mortality, and human resource availability.

### Data Collection

Data collection was done from June to October, 2016, covering a period of 10-years, 2006–2015. Data were collected using customized paper-based collection tools. Sources of data included death registers, inpatient registers, and International Classification of Diseases (ICD-10) report forms. Data collected were deceased's age, sex, underlying cause, and date of death. All death events that occurred at hospitals and recorded were collected. The details of the data collection process and management have been described elsewhere (26).

### Data Management and Analysis

Data were entered into a database developed in EpiData software 3.1. Before entry data were checked for immediate errors. Data entry quality control and edit check was done by taking a percentage of entered data and compared with original data and cleaning up the data by identifying discrepancies. A data acceptable rule was set to remove invalid data, at least a single death was expected within a period of 12 months within a hospital. In case fewer number of deaths were collected, the entire year was discarded from the analysis. No imputation was done to missing age, sex, date, or cause of death. Cleaned dataset was migrated to STATA version 13 for analysis (Stata Corporation College Station, Texas, USA). Data used in this study is a subset of deaths where the underlying cause of death was toxoplasmosis or its co-morbidity. Data were summarized using descriptive statistics and graphical summaries. Annual age standardized mortality rates per 100,000 persons (i.e., a weighted average age-specific mortality rate) was calculated by direct method using the 2012 Population and Housing Census (27) as a reference.

### Ethical Considerations

This study received ethical approval from the Medical Research Coordinating Committee of the National Institute for Medical Research Ref. No. NIMR/HQ/R.8a/Vol. IX/2230. Permissions to access hospital registers and reporting documents were sought from the Ministry of Health, Community Development, Gender, Elderly and Children, and the respective Regional Administrative Secretaries and Hospital Authorities. No informed consent was required in view of the retrospective nature of this study.

## Results

**Figure 1:**
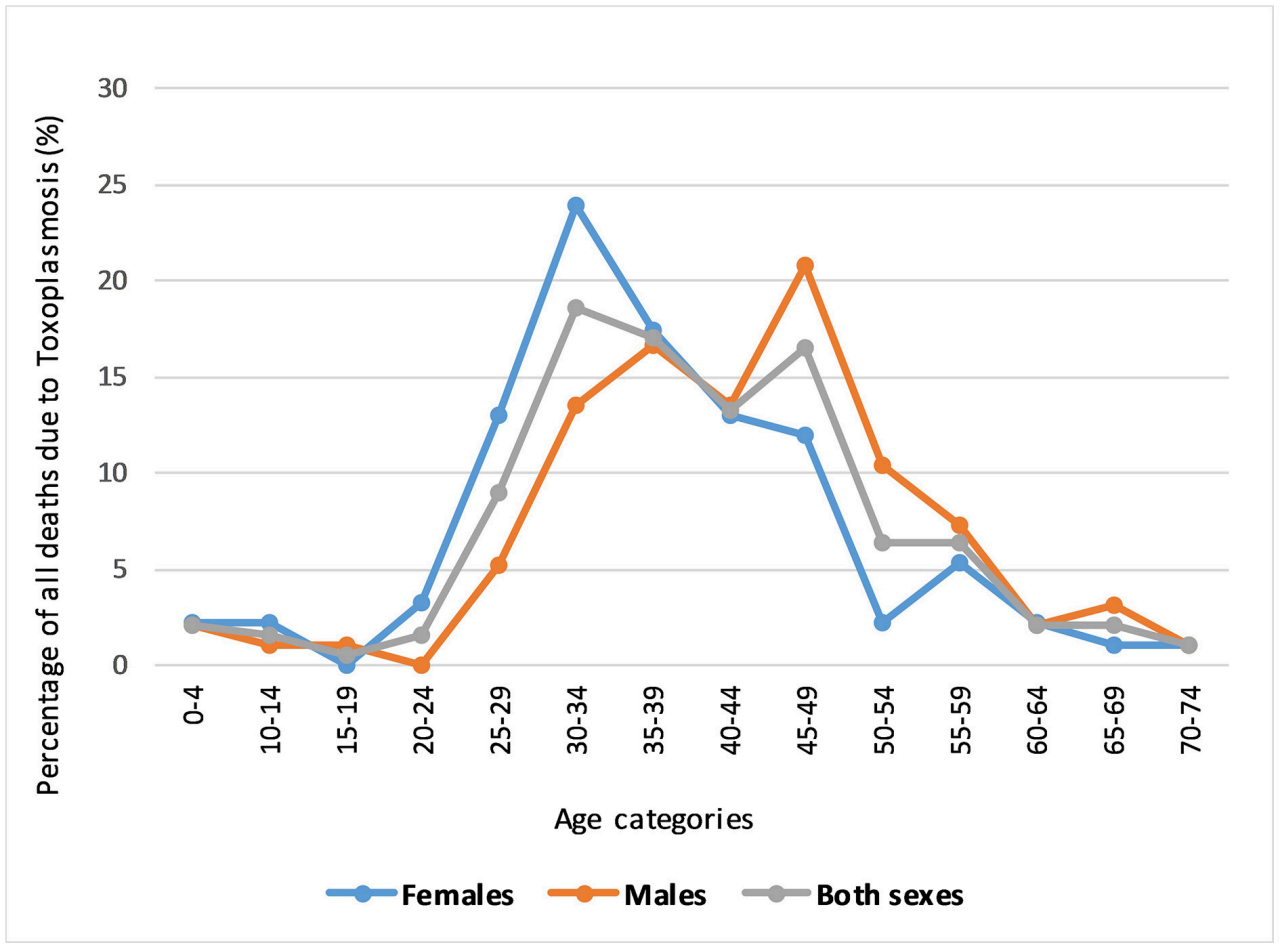
Distribution of death due to toxoplasmosis by age group and sex.

Thirty-seven hospitals were involved in the study. In total of 188 toxoplasmosis associated deaths were identified during the 10-years study period. Of the 188 deaths, males accounted for 51.1% (*n* = 96) and females 48.9% (*n* = 92). Toxoplasmosis associated deaths accounted for 0.08% (188/247,976) of the total death recorded during the period. About two thirds (65.4%) of all toxoplasmosis mortality affected those aged 30–49-years old (Figure 1). The proportion of deaths due to toxoplasmosis were lowest among the young (<24-years) and old age categories (>60-years).

**Figure 2:**
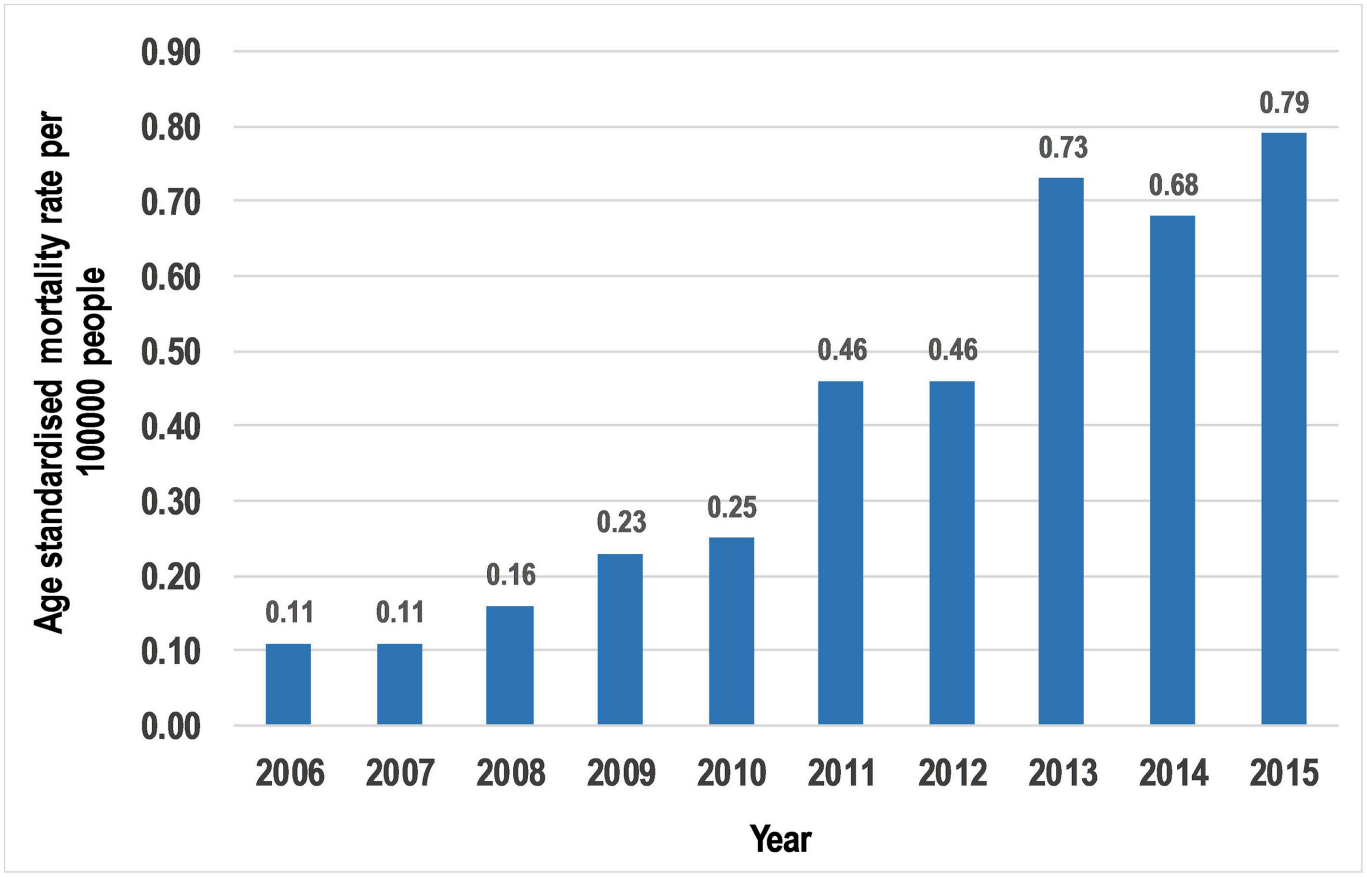
Age standardized mortality rate per 100,000 people.

The number of overall deaths increased from 4 (2.1%) in 2006 to 42 (22.3%) in 2015. Likewise, the annual age-standardized mortality rate per 100,000 population increased from 0.11 in 2006 to 0.79 in 2015 (Figure 2).

**Figure 3:**
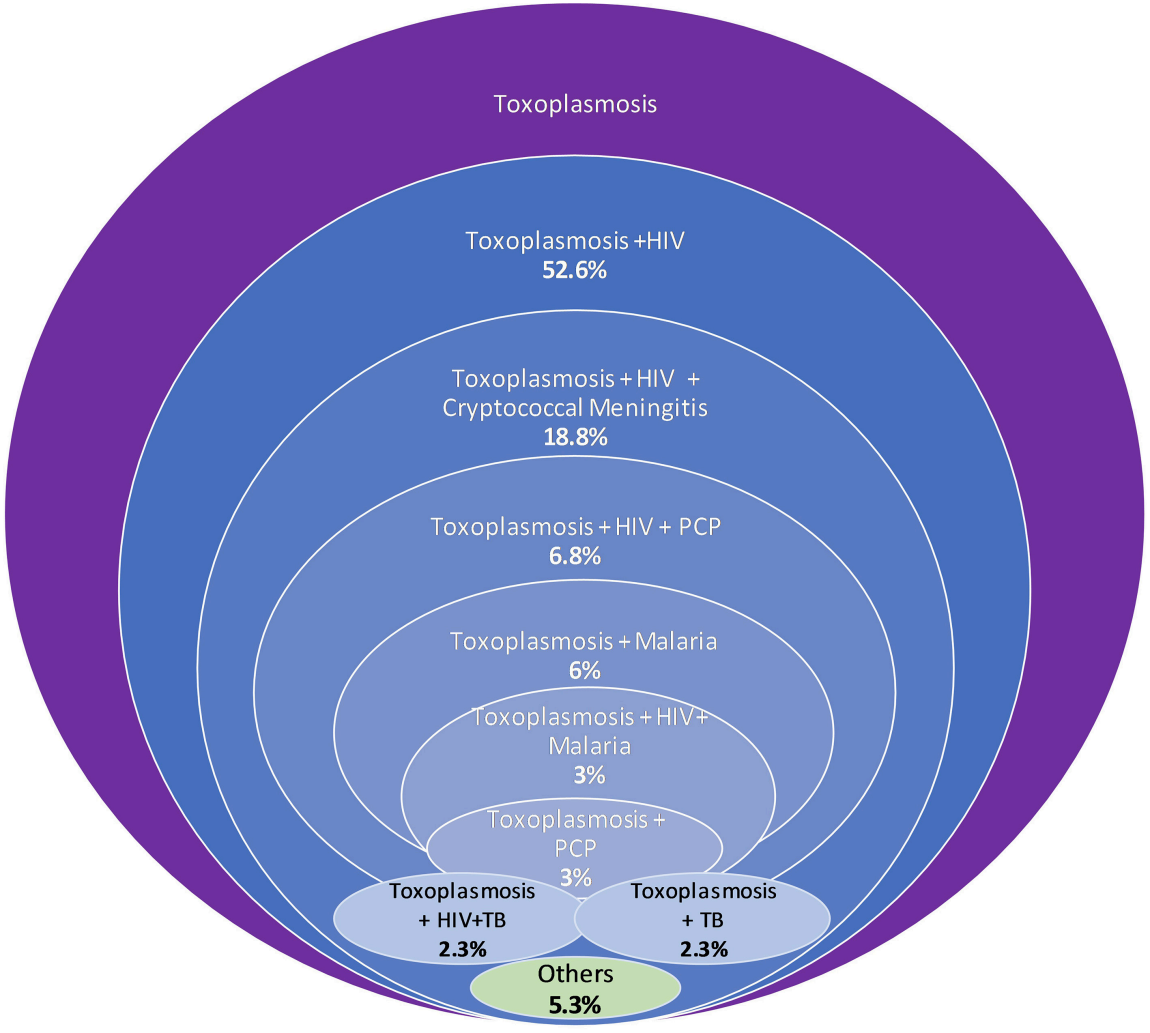
Distribution of number (%) of toxoplasmosis-comorbidities.

Of the total 188 deaths due to toxoplasmosis, 29 (15.4%) were due to cerebral toxoplasmosis and 70.7% (*n* = 133) were associated with other co-morbidities. The medical conditions associated with toxoplasmosis deaths included HIV/AIDS (52.6%), HIV/AIDS+Cryptococcal meningitis (18.8%), HIV+ Pneumocystis pneumonia (6.8%), and malaria (6.0%) (Figure 3). Other medical conditions (5.3%) included malaria+Cryptococcal meningitis, anemia, intoxication, diarrhea, and tetanus. The toxoplasmosis+HIV/AIDS+tuberculosis (TB) co-morbid was found in 2.3% (*n* = 3) of the cases and was similar to that of toxoplasmosis+TB.

**Figure 4:**
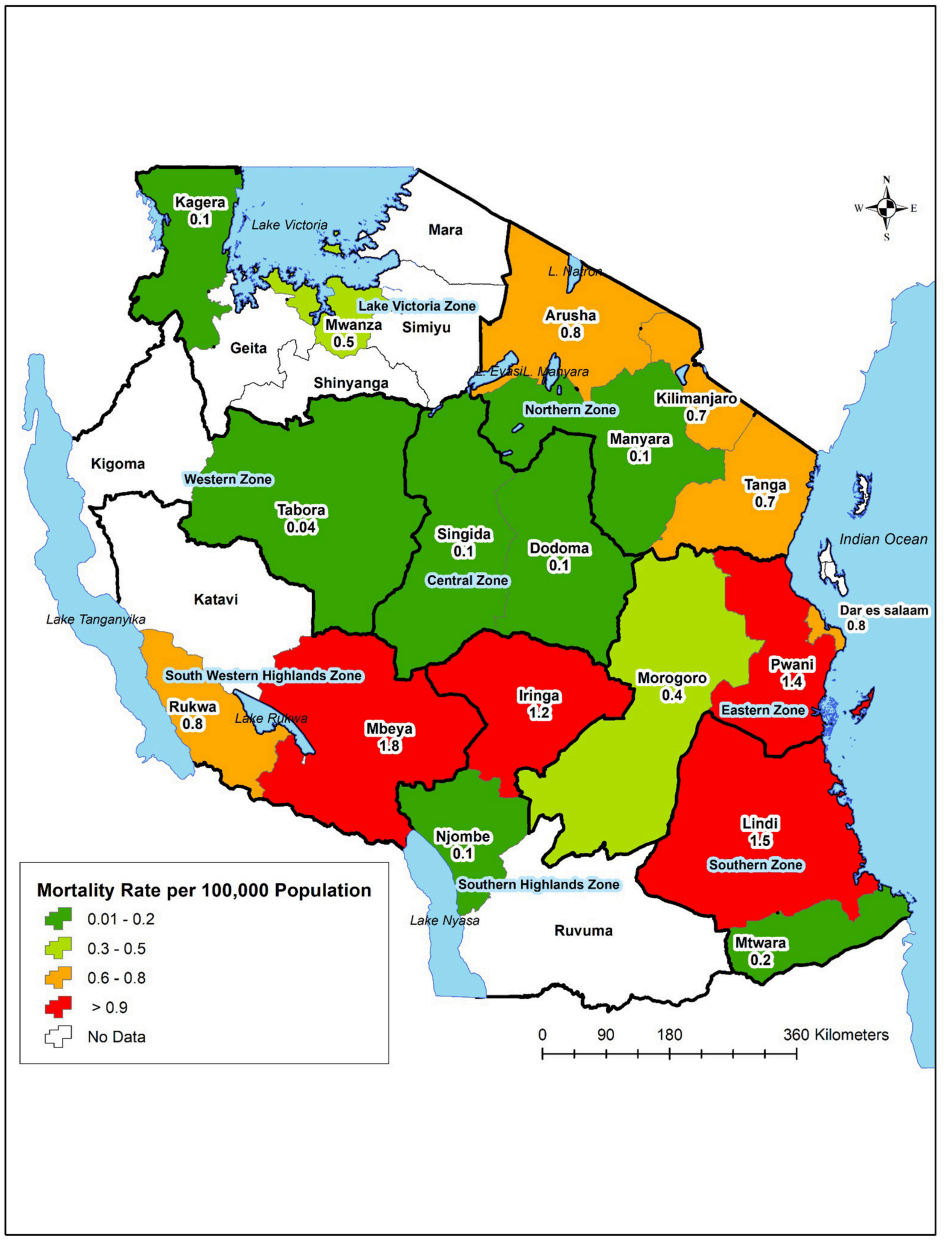
Distribution of deaths due to Toxoplasmosis by geographical region.

The number of deaths varied by geographical region. The eastern (31.4%), south western highlands (21.3%), and northern (20.7%) zones accounted for the majority of deaths. Dar es Salaam (18.62%), Mbeya (17.02%), Pwani (7.98%), Tanga (7.98%), and Mwanza (7.45%) accounted for over half (59.05%) of all deaths due to Toxoplasmosis. However, the mortality rates per 100,000 population were highest in Mbeya (1.8), Lindi (1.5), Pwani (1.4), and Iringa (1.2). Toxoplasmosis deaths were not reported in seven regions, namely Mara, Shinyanga, Simiyu, Geita, Kigoma, Katavi, and Ruvuma (Figure 4).

## Discussion

This paper presents the pattern of mortality due to toxoplasmosis from hospital statistics. On average, 19 hospital deaths due to toxoplasmosis were reported each year in Tanzania. Toxoplasmosis contributed to 0.08% of the total deaths occurring in hospitals of Tanzania during 2006–2015. Most deaths due to toxoplasmosis were reported among the adults. Both females and males were equally affected. Studies elsewhere have reported that in the majority of the human populations, the seroprevalence *T. gondii* may vary by sex (21, 28). Women have been described to be more often affected than men (29). Moreover, pregnant women are known to be at higher risk for *T. gondii* primary infections (30). In this study, most deaths associated with Toxoplasmosis occurred among the middle-aged groups, although peak much earlier in women than in men. Higher risk in young women could be attributed to their day-to-day involvement with domestic activities including house cleaning, which might expose them to infected animals or feces but also the peak of the reproductive age. Although similar mortality studies are lacking, some studies have shown that the prevalence of *T. gondii* increases with age (16, 21, 28, 31).

Three quarters of the deaths due to toxoplasmosis in our current study were associated with HIV-co-morbidity. The age peak of deaths due to toxoplasmosis, for both sexes, are the same age categories reported to have the highest HIV/AIDS prevalence in Tanzania (32). *T. gondii* infection is known to be common among people who have immunosuppression such as those with HIV virus (21, 33). Studies have reported that toxoplasmosis is the leading cause of central nervous system lesions in AIDS patients (34, 35). In this study, cerebral toxoplasmosis accounted for 15.4% of the toxoplasmosis deaths. Cerebral toxoplasmosis has been reported to account for 10% of deaths due to AIDS in West Africa (25). Other studies have indicated that cerebral toxoplasmosis is one of the major opportunistic infections that occurs in 5–40% of patients with AIDS (36, 37). However, a most recent study in Uganda has reported a case of primary cerebral toxoplasmosis in an HIV-negative individual adult; indicating that HIV/AIDS may not be a prerequisite for cerebral toxoplasmosis (38).

The regional variation in the number of deaths due to toxoplasmosis was observed in this study. Higher mortality rates per population were reported in regions of the south-western highlands, southern and eastern zones. Mbeya Region is known to have higher HIV seroprevalence (32). The higher mortality rate of Toxoplasmosis in Lindi could not be associated with HIV as the prevalence of HIV in this region is relatively low (32). Other factors are likely to play a role to the higher mortality rates in Lindi. In the southern regions of Tanzania, including Lindi, rodent hunting and consumption are popular (http://haliproject.org/blog/2013/5/9/roasted-mice), and are likely to put the community at risk of acquiring Toxoplasma infection. A prevalence of 2.17% of *T. gondii* in rodents has been reported in Tanzania (39). Variations in the burden of Toxoplasma infection have been attributed to several factors including environmental, socio-economic, quality of water sanitation, eating habits, and being HIV positive (20). Most of the deaths due to toxoplasmosis were also reported from hospitals located in the largest cities in Tanzania. Like many other large cities in East Africa, there is a high preference for consumption of barbeque (*nyama choma)* (40). *Nyama choma* which is usually undercooked pose the likelihood of consumers ingesting the parasites from the animal meat, especially in this part of the world where the awareness of Toxoplasmosis is low (20).

Like in mortality, geographical variations in the prevalence of *T. gondii* has been reported to be attributed to environmental factors such as food production system practices, water treatment, topography, climate, hygiene, occupation, and culinary practices (18, 23). In a study in north-eastern Tanzania, the seroprevalence of toxoplasma infection was significantly higher amongst abattoir workers and individuals who keep livestock (18). In another study in north-western Tanzania, higher seroprevalence of *T. gondii* was found among women from urban than from rural communities; and that employed/business women were more likely to be infected than peasants (18). Hygienic measures such as washing fruits and vegetables, avoiding consumption of raw and undercooked meat, and washing hands after gardening or handling cats have been described to reduce the transmission of the parasite (41). However, caution must be taken as in some areas water used for washing may be contaminated with toxoplasmosis (42). It is important that studies on risk factors associated with Toxoplasmosis in different part of the country are conducted to provide evidence on its current distribution and awareness to guide appropriate interventions.

The findings of this study are likely to have some limitations due to its design and data sources. Only deaths that occurred in hospitals and the patients tested for toxoplasmosis are reported here. This might miss a proportion of those not seeking health care from hospitals or not tested for the infection. An increasing trend in the number of deaths due to toxoplasmosis observed during the 10-years period under review could be due to a true increase in mortality or due to an improvement in the ability to diagnose the disease as a cause of death. An improvement in the completeness and quality of death registration and storage due to investment in data quality could also influence the increasing trend of deaths due to toxoplasmosis in recent years. In a parallel study, an increase in completeness and availability of death records from 2006 to 2015 has been observed (43). It is therefore critical to strengthen hospital capacities in clinical microbiology as well as data management to be able to timely detect, manage, and respond to the increasing risk and burden of toxoplasmosis in the country.

## Conclusion

Annually, 19 deaths due to toxoplasmosis are reported in hospitals of Tanzania, with the number increasing substantially in recent years. Most deaths due to toxoplasmosis affect the adult age category and were associated with HIV/AIDS. Appropriate interventions, including strengthening diagnostic capacities are needed to improve the management of the diseases in our health care facilities. Being a zoonosis, the results of the present study therefore, advocate for implementation of preventive measures through a one health approach.

## Author Contributions

LM and SR participated in study design and acquisition of data. CK, SR, LM, and EK performed the statistical analysis. LM drafted the manuscript. All authors read and approved the final draft of the manuscript.

### Conflict of Interest Statement

The authors declare that the research was conducted in the absence of any commercial or financial relationships that could be construed as a potential conflict of interest. The handling editor and reviewer OV declared their involvement as co-editors in the Research Topic, and confirm the absence of any other collaboration.
